# Clinical characteristics and risk factors for in-hospital mortality of COVID-19 patients in Hubei Province: A multicenter retrospective study

**DOI:** 10.1016/j.ijcha.2024.101574

**Published:** 2024-11-30

**Authors:** Wu He, Gen Li, Ke Xu, Bo Yu, Yang Sun, Kaineng Zhong, Da Zhou, Yongcui Yan, Junfang Wu, Dao Wen Wang

**Affiliations:** a Division of Cardiology, Department of Internal Medicine, Tongji Hospital, Tongji Medical College, Huazhong University of Science and Technology; Hubei Key Laboratory of Genetics and Molecular Mechanisms of Cardiological Disorders, Wuhan 430030, China; b Health Commission of Hubei Province, Wuhan 430079, China

**Keywords:** COVID-19, SARS-CoV-2, In-hospital mortality, Clinical characteristics, Risk factors

## Abstract

**Background:**

Coronavirus disease (COVID-19) remains one of the most significant factors threatening public health security worldwide. The COVID-19 pandemic has been ongoing for more than 3 years; however, there are few studies on the clinical characteristics and mortality risk factors in patients with COVID-19 based on comprehensive data from multiple centers.

**Methods:**

A total of 53,030 patients with confirmed COVID-19 from 138 hospitals in Hubei Province were included in this study. We compared the clinical characteristics between survivors and non-survivors and analyzed the risk factors for in-hospital mortality.

**Results:**

Among the 53,030 patients with COVID-19, 49,320 (93.0 %) were discharged, and 3,710 (7.0 %) died during hospitalization. Cardiovascular disease was the most common comorbidity, followed by endocrine and digestive diseases. Male sex, >65-year-old, and high diastolic blood pressure, a series of abnormal laboratory test indicators and hyponatremia, hypokalemia, acute respiratory distress syndrome, shock, solid tumor, hematological tumor, and insulin use were independent risk factors for in-hospital mortality of patients with COVID-19. In addition, male sex, older age, and higher disease severity were associated with increased mortality in patients with COVID-19.

**Conclusion:**

Patients with early COVID-19 in Hubei Province had high mortality and a high proportion of severe cases and initial comorbidities. Cardiovascular disease was the most common comorbidity in patients with COVID-19. Male sex, older age, comorbidities, and abnormal laboratory data have been identified as independent risk factors for in-hospital mortality in patients with COVID-19. Therefore, there should be an increased focus on patients with COVID-19 with these risk factors.

## 1 Introduction

In December 2019, a novel coronavirus disease (COVID-19) caused by severe acute respiratory syndrome coronavirus 2 (SARS-CoV-2) first appeared in Wuhan, Hubei Province, China, and rapidly spread worldwide[1], [2]. Confirmed cases of COVID-19 have exceeded 775 million by 28 July 2024, including more than 7 million deaths. Although COVID-19 vaccines and oral COVID-19 antiviral drugs have been tested[3], [4], controlling the progression and mortality of COVID-19 has not been clinical achieved and remains a tremendous challenge[5].

Clinical characteristics and mortality risk factors of patients with COVID-19 have been studied in many countries[2], [6], [7], [8]. However, relatively few multicenter and large-sample studies have been conducted[9]. Additionally, the risk factors associated with in-hospital mortality among patients with COVID-19 of differing severity have not been systematically identified. The majority of patients with COVID-19 in China were treated in hospitals in Hubei Province during the outbreak period[10], [11]. Therefore, systematic data of patients with early COVID-19 in Hubei Province are of the highest quality and most reliable for elucidating the characteristics and risk factors associated with COVID-19 mortality.

To clarify the clinical characteristics and mortality risk factors of patients with COVID-19 in Hubei from 2019 to 2021, we conducted a multicenter retrospective study of 53,030 patients with COVID-19 (discharged or deceased) in 138 hospitals in Hubei, China. We examined the risk factors for mortality in patients with COVID-19 of varying severity. Furthermore, we investigated the survival of patients with COVID-19 of different sexes, ages, and severity levels. This study is part of a larger study on the genetics and treatment of COVID-19, the results of which will guide clinical decision-making and the rational allocation of health resources.

## 2 Methods

### 2.1 Study design and patient information

In this cohort study, we collected information on inpatients diagnosed with confirmed SARS-CoV-2 infection from 138 hospitals in the Hubei Province. According to China and WHO interim guidance, all patients diagnosed with COVID-19 between Dec 29, 2019 (i.e., when the first patients were admitted) and Aug 31, 2021, were screened, and patients who had died or were discharged were included in the study. For laboratory confirmation of SARS-CoV-2 infection, nasal and pharyngeal swabs must show at least two consecutive positive results from high-throughput sequencing or real-time reverse transcriptase–polymerase chain reaction assays. Participants < 18-year-old were excluded. In addition, due to irregularities in the uploading of patient information by some doctors in some hospitals, important information of some patients was not uploaded into the database, such as reverse transcriptase-polymerase chain reaction results of SARS-CoV-2, related basic information, laboratory results and treatment information. Patients without such important information were also excluded. CURB-65 score is a widely used score system to guide the treatment of community-acquired pneumonia (CAP), which consists of five easily acquired parameters (confusion, blood urea nitrogen, respiratory rate, blood pressure and age)[12]. Patients are stratified into five risk categories.

This study included 53,030 inpatients > 18-year-old who were diagnosed with COVID-19. Patients were divided into death and survival groups based on their discharge outcomes. The patients were classified into mild, moderate, severe, and critical types based on the disease severity. The classification was based on the diagnosis and treatment of COVID-19 guidelines (sixth version) published by the National Health Commission of China.

### 2.2 Data Sources

This retrospective study used registry data of COVID-19 inpatients in Hubei Province from 2019 to 2021. Desensitized data (excluding name, address, and contact information) for all patients hospitalized for COVID-19 infection were uploaded to the public electronic medical record system of the Health Commission of Hubei Province, constituting a database. Only patients hospitalized for COVID-19 infection were included in this database. All the data for this study were collected from the electronic medical record database. The study was conducted in accordance with the principles of the Declaration of Helsinki and was approved by the Research Ethics Committee of Tongji Medical College (no. TJ-IRB20210138). Due to the retrospective nature of this study and the anonymity of its participants, the ethics committee waived the requirement for written informed consent.

### 2.3 Data collection and endpoint definitions

In most cases, we extracted epidemiological, demographic, clinical, and laboratory data from electronic medical records at the time of admission. These records were independently examined by three researchers to obtain the basic information and clinical data of each patient. The collected information included age, sex, comorbidities, exposure history, oxygen support during hospitalization, vital signs (temperature, respiratory rate, pulse rate, and systolic and diastolic blood pressures [SBP and DBP]), serum laboratory tests (including blood routine tests, blood chemical variables, procalcitonin, coagulation function tests), therapeutic strategy during hospitalization, and outcomes. The laboratory biochemical test results were mainly checked by double-antibody sandwich method of chemiluminescence technique based on Roche platform. To reflect the initial status of COVID-19 patients, the laboratory test results included in the analysis were based on the initial test results of COVID-19 patients during hospitalization. The treatment information of COVID-19 patients after admission was determined according to the executed temporary and long-term medical orders. During this retrospective study, all-cause mortality during hospitalization was the primary endpoint of concern. The primary endpoint was also based on electronic medical records within hospital data. The hospitalization time (days) was calculated by the time of death or discharge from hospital minus the time of admission.

### 2.4 Statistical analysis and mapping

Statistical analysis and mapping were performed using SPSS (version 24.0; IBM, Armonk, NY, USA), R (version 4.1.1; R Foundation for Statistics Computing, Vienna, Austria), and GraphPad Prism (version 9.0; San Diego, CA, USA). The distribution and homoscedasticity of each dataset were tested using D'Agostino’s and Pearson’s omnibus normality. Continuous normally distributed data were reported as mean (deviation) and compared using the student’s *t*-test. Continuous non-normally distributed data were reported as medians (interquartile range, IQR) and compared using the Wilcoxon rank-sum test. Categorical data are presented as n (percentage) and were compared using the Chi-square test, Fisher’s exact test, and the Cochran–Mantel–Haenszel test, as appropriate. The optimal calliper width was set at 0.05.

Univariate and multivariate Cox regression models were used to determine the risk factors for death during hospitalization and are displayed as hazard ratios (HRs) and 95 % confidence intervals (CIs). Variables with P < 0.05 in the univariate analysis or with important clinical significance were considered candidate variables in the multivariable analysis. Survival analysis methods, including Kaplan–Meier curves and Cox proportional hazard model analyses, were used to compare the time of endpoint events in different subgroups.

### 2.5 Patient and public involvement

Patients and the public were not involved in the design or conduct of the study. However, we plan to disseminate our main research findings to clinicians and patients at national and district levels, as well as in national and international conferences.

## 3 Results

### 3.1 Demographic and clinical characteristics

**Fig. 1:**
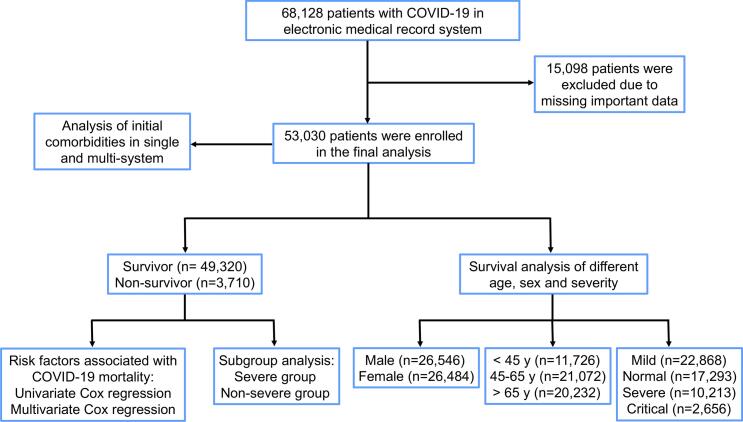
The flowchart of study design.

This study included 68,128 patients with COVID-19 from 138 hospitals in Hubei Province before Aug 31, 2021. Of these, 15,098 (22.2 %) patients were excluded due to the lack of critical data. Ultimately, 53,030 inpatients were included in the analysis. Among them, 49,320 (93.0 %) were discharged and 3,710 (7.0 %) died during hospitalization (Fig. 1). The median age of the patients was 59 years (IQR, 47–70), and 26,546 (50.1 %) patients were male (Table 1). More than half of the patients had initial comorbidities, of which hyperlipidemia (52.3 %) and hypertension (51.4 %) were the most common, followed by diabetes (9.3 %) and coronary heart disease (6.1 %). The numbers of severe and critical patients were 10,213 (19.3 %) and 2656 (5.0 %), respectively (Table 1). The median time from admission to discharge was 13 days (IQR: 7–20), whereas the median time to death was 9 d (IQR: 4–17; Supplementary Table S1). The baseline vital signs and laboratory results (routine blood test results, blood biochemistry profile, infection-related indicators, and coagulation function) of the surviving and deceased patients are also shown in Table 1. Additionally, the use of systemic corticosteroids and intravenous immunoglobulin (IVIg) was significantly different between non-survivors and survivors, as was the use of mechanical ventilation, extracorporeal membrane oxygenation (ECMO), and continuous renal replacement therapy (CRRT) (Table 1). The proportion of patients with sepsis and acute respiratory distress syndrome (ARDS) during hospitalization was also significantly different between the two groups (Table 1). In addition, a higher proportion of the non-survivors had thrombotic complications (include pulmonary embolism, deep venous thrombosis, peripheral arterial ischaemia, myocardial infarction, stroke or extracorporeal circuit thrombosis) compared to the survivors(Table 1). Among non-severe patients, the proportion of non-survivors with thrombotic complications was higher than that of survivors (Supplementary Table S1). Among severe patients, the proportion of non-survivors with thrombotic complications was also higher than that of survivors (Supplementary Table S2).

**Table 1:** Clinical characteristics of patients with COVID-19.

	**Total (n = 53030)**	**Survivor (n = 49320)**	**Non-survivor (n = 3710)**	**P**
Gender: Male (%)	26,546 (50.1)	24,282 (49.2)	2264 (60.5)	<0.001
Age, years	59 (47–70)	58 (46–69)	71 (63–80)	<0.001
Age ≥ 65 years (%)	20,232 (38.2)	17,622 (35.7)	2610 (70.4)	<0.001
Age＜65 years (%)	32,798 (61.8)	31,698 (64.3)	1100 (29.6)	<0.001
Hospitalization time (days)	13 (7–20)	13 (7–20)	9 (4–17)	<0.001
Severity (%)				
Mild	22,868 (43.1)	21,981 (44.6)	887 (23.9)	0.303
Normal	17,293 (32.6)	16,592 (33.6)	701 (18.9)	0.086
Severe	10,213 (19.3)	9085 (18.4)	1128 (30.4)	<0.001
Critical	2656 (5.0)	1662 (3.4)	994 (2.7)	<0.001
**Vital signs**				
Systolic blood pressure (mmHg)	121 (105–133)	121 (106–123)	120 (92–132)	<0.001
Diastolic blood pressure (mmHg)	80 (72–93)	80 (72–93)	80 (71–98)	0.7514
Respiratory rate (times per minute)	20 (19–20)	20 (19–20)	20 (19–21)	<0.001
Pulse rate (times per minute)	80 (75–83)	80 (75–88)	83 (76–95)	<0.001
Temperature (°C)	36.5 (36.3–36.8)	36.5 (36.3–36.8)	36.7 (36.4–36.8)	<0.001
**Original comorbidities (%)**				
Hypertension (%)	9736 (18.4)	8977 (18.2)	759 (20.5)	<0.001
Diabetes (%)	4922 (9.3)	4535 (9.2)	387 (10.4)	0.013
Hyperlipidemia (%)	27,713 (52.3)	25,475 (51.7)	2238 (60.3)	<0.001
Coronary heart disease (%)	3213 (6.1)	2953 (6.0)	260 (7.0)	0.013
COPD (%)	1164 (2.2)	1071 (2.2)	93 (2.5)	0.199
Heart failure (%)	1418 (2.7)	1168 (2.4)	250 (6.7)	<0.001
Arrhythmia (%)	1353 (2.6)	1244 (2.5)	109 (2.9)	0.135
CKD (%)	1045 (2.0)	772 (1.6)	273 (7.4)	<0.001
Solid tumor (%)	970 (1.8)	857 (1.8)	113 (3.0)	<0.001
Hematological tumor (%)	81 (0.2)	64 (0.1)	17 (0.5)	<0.001
**Laboratory results**				
**Routine blood test**				
Red blood cell count, ×10^12^/L	4.17 (3.78–4.57)	4.18 (3.79–4.57)	4.10 (3.65–4.51)	<0.001
White cell count, ×10^9^/L	5.82 (4.61–7.53)	5.79 (4.60–7.43)	6.68 (5.01–9.62)	<0.001
Hemoglobin g/L	126 (114–138)	126 (115–138)	123 (109–137)	<0.001
Neutrophil count, ×10^9^/L	3.64 (2.7–5.17)	3.59 (2.68–5.03)	4.64 (3.18–8.02)	<0.001
Monocyte count, ×10^9^/L	0.41 (0.3–0.54)	0.41 (0.30–0.54)	0.40 (0.29–0.55)	0.034
Platelet count, ×10^9^/L	201 (160–240)	202 (162–241)	184 (134–230)	<0.001
**Blood biochemistry**				
Alanine aminotransferase, U/L	21.3 (14–35.9)	21 (14–35)	23 (15–40)	<0.001
Aspartate aminotransferase, U/L	22.0 (17–31.5)	22 (17–31)	26 (19–42)	<0.001
Lactate dehydrogenase, U/L	190 (156–248)	188 (155–243)	232 (172–378)	<0.001
Total bilirubin, umol/L	10.1 (7.4–13.9)	10.1 (7.4–13.8)	10.7 (7.8–15.3)	<0.001
Total protein, g/L	67 (62.3–71.8)	67.1 (62.4–71.9)	65.8 (60.3–70.9)	<0.001
Globulin, g/L	28.0 (24.4–31.8)	27.9 (24.4–31.7)	29.2 (25.2–33.5)	<0.001
Albumin, g/L	38.7 (34.7–42.6)	38.9 (34.9–42.8)	35.9 (31.1–40.5)	<0.001
Alkaline phosphatase, U/L	67 (54–85)	67 (54–85)	71 (55–92)	<0.001
Total cholesterol, mmol/L	4.12 (3.86–4.86)	4.14 (3.48–4.88)	3.84 (3.17–4.59)	<0.001
Triglyceride, mmol/L	1.3 (0.96–1.85)	1.30 (0.96–1.85)	1.35 (0.97–1.93)	<0.001
LDL, mmol/L	2.43 (1.91–3.03)	2.44 (1.93–3.04)	2.25 (1.71–2.83)	<0.001
HDL, mmol/L	1.07 (0.88–1.31)	1.08 (0.89–1.31)	0.98 (0.79–1.21)	<0.001
Creatinine, μmol/L	66 (55–81)	66 (55–80)	71 (57–91)	<0.001
Blood urea nitrogen, mmol/L	4.6 (3.6–5.9)	4.5 (3.6–5.8)	5.5 (4.0–8.4)	<0.001
eGFR, mL/min	99.73 (85.8–114.1)	100.0 (86.6–114.5)	95.2 (73.8–110.7)	<0.001
Sodium, mmol/L	139.9 (137.3–141.8)	139.5 (137.4–141.8)	139.4 (136.7–142.0)	0.002
Potassium, mmol/L	4.02 (3.7–4.36)	4.01 (3.70–4.35)	4.04 (3.68–4.43)	0.003
Calcium, mmol/L	2.24 (2.11–2.36)	2.24 (2.12–2.37)	2.16 (2.02–2.32)	<0.001
Lactic acid, mmol/L	1.7 (1.22–2.30)	1.7 (1.2–2.3)	1.9 (1.4–2.6)	<0.001
**Coagulation function**				
APTT, s	31.3 (27.6–35.4)	31.3 (27.5–35.3)	32.5 (28.3–37.3)	<0.001
Prothrombin time, s	12.3 (11.0–13.5)	12.3 (11.0–13.5)	12.9 (11.4–14.3)	<0.001
Prothrombin activity, %	96.0 (85.5–106.2)	96 (86–107)	91 (78–102)	<0.001
Thrombin time, s	16.5 (15.3–17.8)	16.5 (15.3–17.8)	16.6 (15.3–18.1)	0.001
International normalized ratio	1.01 (0.91–1.11)	1.00 (0.91–1.10)	1.06 (0.95–1.09)	<0.001
Fibrinogen, mg/L	3.32 (2.58–4.29)	3.30 (2.58–4.28)	3.48 (2.62–4.59)	<0.001
D-dimer, mg/L	0.41 (0.24–0.88)	0.46 (0.24–0.87)	0.59 (0.29–1.16)	<0.001
Glucose, mmol/L	5.68 (5.0–7.11)	5.64 (4.99–7.00)	6.30 (5.22–8.73)	<0.001
**Treatment**				
Corticosteroids (%)	4300 (8.1)	3396 (6.9)	904 (24.4)	<0.001
Intravenous immunoglobin (%)	5630 (10.6)	4490 (9.1)	1140 (30.7)	<0.001
Mechanical ventilation (%)	1818 (3.4)	728 (1.5)	1090 (29.4)	<0.001
ECMO (%)	196 (0.37)	144 (0.29)	52 (1.4)	<0.001
CRRT (%)	382 (0.72)	194 (0.39)	188 (5.1)	<0.001
**Complications**				
Sepsis (%)	400 (0.75)	235 (0.48)	165 (4.4)	<0.001
ARDS (%)	551 (1.04)	277 (0.56)	274 (7.39)	<0.001
Thrombotic complications (%)*	2956 (5.57)	2614 (5.30)	342 (9.22)	<0.001

Data were presented as median and interquartile range (Q1-Q3). Keys: COPD, chronic obstructive pulmonary disease; CKD, chronic kidney disease; LDL, low-density lipoprotein; HDL, high-density lipoprotein; eGFR, estimated glomerular filtration rate; APTT, activated partial thromboplastin time; ECMO, extracorporeal membrane oxygenation; CRRT, continuous renal replacement therapy; ARDS, acute respiratory distress syndrome.

* Thrombotic complications include pulmonary embolism, deep venous thrombosis, peripheral arterial ischaemia, myocardial infarction, stroke or extracorporeal circuit thrombosis.

### 3.2 Initial comorbidities in single and multi-system

**Fig. 2:**
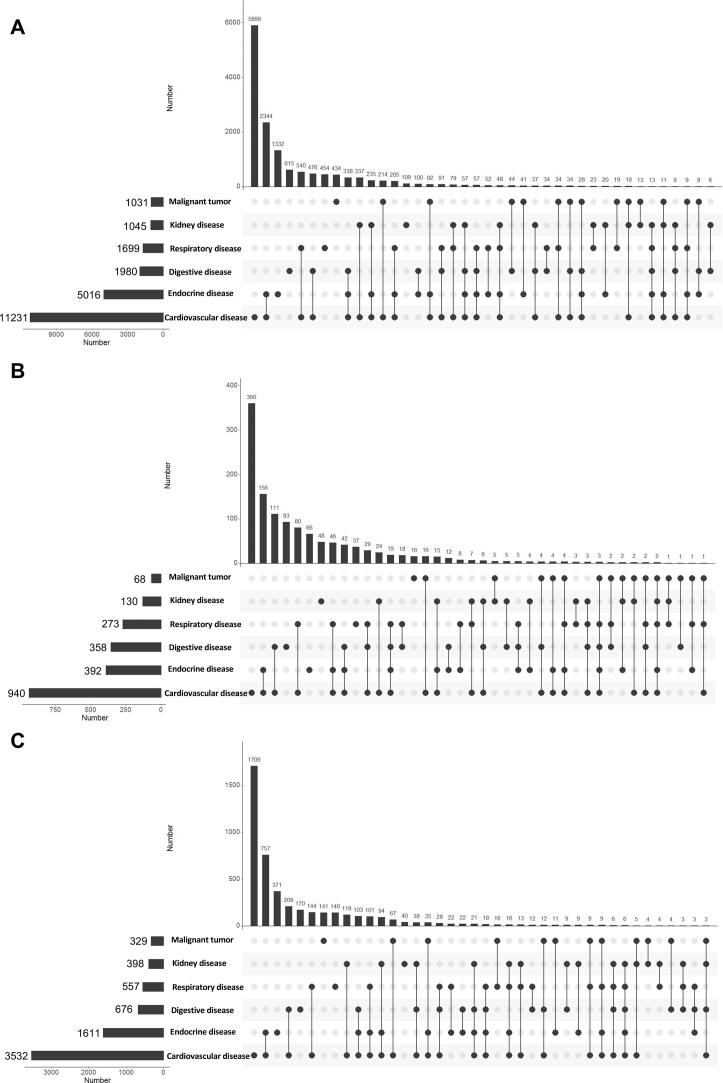
Initial comorbidities in patients with COVID-19. (A) Initial comorbidities in all patients with COVID-19. (B) Initial comorbidities in non-survivors with COVID-19. (C) Initial comorbidities in severe patients with COVID-19.

We compared the initial comorbidities of the different systems (cardiovascular diseases: hypertension, coronary heart disease, chronic heart failure, and arrhythmia; respiratory diseases: chronic bronchitis, and chronic obstructive pulmonary disease; digestive diseases: chronic gastritis, gastric ulcer, liver cirrhosis, inflammatory bowel disease, and pancreatitis; kidney diseases: chronic kidney disease; endocrine diseases: diabetes and thyroid disease; malignant tumor: solid tumor and hematological tumor) in all patients, non-survivors, and severe patients (Figure 2). Cardiovascular disease (n = 11,231) was the most common comorbidity in all patients, followed by endocrine (n = 5,016) and digestive diseases (n = 1,980) (Figure 2A). The number of patients with cardiovascular disease complicated by endocrine disease (n = 2,344) was the highest among all patients with two comorbidities (Figure 2A). Similar results were observed in non-survivors and critically ill patients (Figure 2B and 2C).

### 3.3 Risk factors of in-hospital mortality in patients with COVID-19

**Fig. 3:**
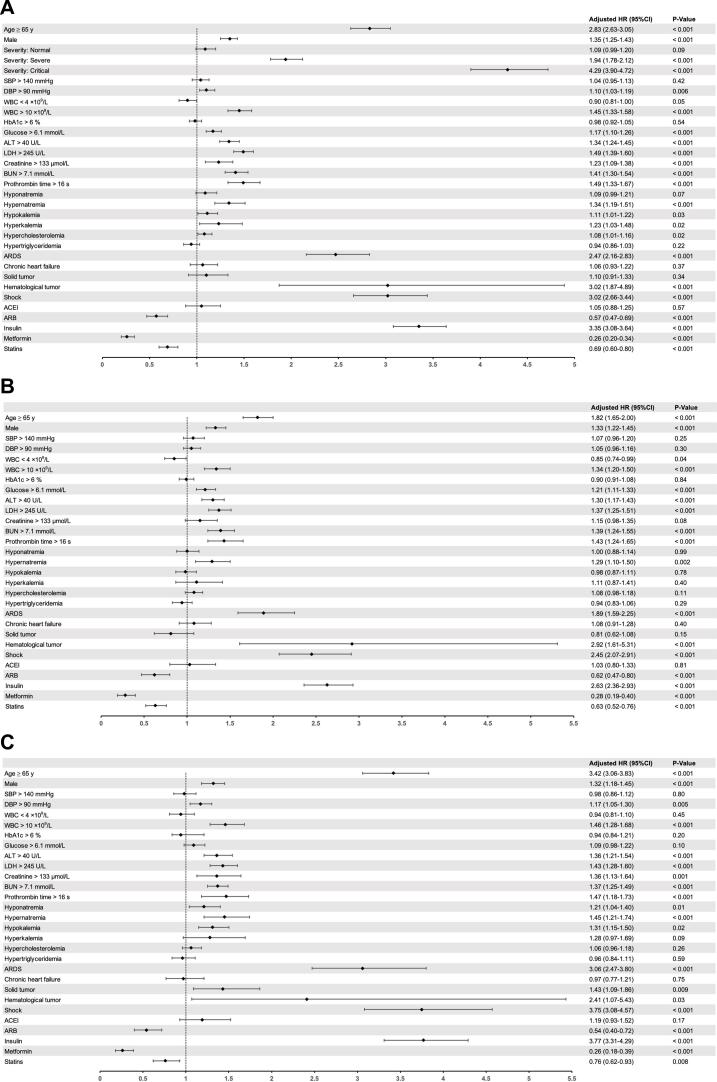
Hazard ratio for risk factors associated with in-hospital mortality. (A) In all patients with COVID-19. (B) In non-severe patients with COVID-19. (C) In severe patients with COVID-19.

To evaluate the risk factors for in-hospital mortality for patients with COVID-19, univariate Cox regression was performed on 64 candidate variables based on the differences between groups and their clinical significance (Table 2). To conclude, through the stepwise forward Cox regression model, 17 variables were identified as risk factors for COVID-19 mortality in the entire cohort from the 29 variables included in the multivariate regression (Table 2, Fig. 3A). Notably, age > 65 years was identified as an independent risk factor for mortality (adjusted hazard ratio [HR], 3.42; 95 % confidence interval [CI], 3.06–3.83; P < 0.001). In addition, important independent risk factors for in-hospital mortality in patients with COVID-19 were male sex, DBP > 90 mmHg, increased white blood cell (WBC, >10 × 10^9^ /L), blood glucose (>6.1 mmol/L), alanine aminotransferase (ALT, >40 U/L), lactate dehydrogenase (LDH, >245 U/L), creatinine (Cr, >133 μmol/L), and blood urea nitrogen (BUN, >7.1 mmol/L) levels, and increased prothrombin time (PT, > 16 s) as well as hyponatremia, hypokalemia, ARDS, shock, solid tumors, hematological tumors, and insulin use (Fig. 3A). Moreover, we used univariate and multivariate Cox regression to analyze the risk factors for in-hospital mortality in patients with non-severe and severe COVID-19 (Supplementary Tables S1-S4), and the independent risk factors were similar to the overall results (Fig. 3B and 3C).

**Table 2:** Risk factors associated with in-hospital mortality in all COVID-19 patients.

**Characteristics**	**Unadjusted HR (95 %CI)**	**P**	**Adjusted HR (95 %CI)**	**P**
Age, years				
<65	1 (ref)		1 (ref)	
≥ 65	3.46 (3.22–3.71)	< 0.001	2.83 (2.63–3.05)	< 0.001
Sex				
Female	1 (ref)		1 (ref)	
Male	1.54 (1.45–1.64)	< 0.001	1.35 (1.25–1.43)	< 0.001
Severity				
Light	1 (ref)		1 (ref)	
Moderate	1.05 (0.95–1.16)	0.33	1.09 (0.99–1.20)	0.09
Severe	2.56 (2.34–2.79)	< 0.001	1.94 (1.78–2.12)	< 0.001
Critical	9.12 (8.32–9.99)	< 0.001	4.29 (3.90–4.72)	< 0.001
Systolic blood pressure (mmHg)				
≤ 140	1 (ref)		1 (ref)	
> 140	1.09 (1.00–1.19)	0.05	1.04 (0.95–1.13)	0.42
Diastolic blood pressure (mmHg)				
≤ 90	1 (ref)		1 (ref)	
> 90	1.16 (1.08–1.24)	< 0.001	1.10 (1.03–1.19)	0.006
White blood cell count, ×10^9^/L				
4–10	1 (ref)		1 (ref)	
< 4	0.82 (0.74–0.91)	< 0.001	0.90 (0.81–1.00)	0.05
>10	2.85 (2.64–3.08)	< 0.001	1.45 (1.33–1.58)	< 0.001
HbA1c, %				
≤ 6	1 (ref)		1 (ref)	
> 6	1.07 (1.00–1.15)	0.04	0.98 (0.92–1.05)	0.54
Glucose, mmol/L				
≤ 6.1	1 (ref)		1 (ref)	
> 6.1	1.78 (1.67–1.90)	< 0.001	1.17 (1.10–1.26)	< 0.001
Alanine aminotransferase, U/L				
≤ 40	1 (ref)		1 (ref)	
> 40	2.02 (1.88–2.18)	< 0.001	1.34 (1.24–1.45)	< 0.001
Lactate dehydrogenase, U/L				
≤ 245	1 (ref)		1 (ref)	
> 245	2.40 (2.25–2.56)	< 0.001	1.49 (1.39–1.60)	< 0.001
eGFR, mL/min				
≥ 90	1 (ref)		1 (ref)	
< 90	1.55 (1.45–1.65)	< 0.001	0.99 (0.92–1.07)	0.80
Creatinine, μmol/L				
≤ 133	1 (ref)		1 (ref)	
> 133	3.13 (2.82–3.47)	< 0.001	1.23 (1.09–1.38)	< 0.001
Blood urea nitrogen, mmol/L				
≤ 7.1	1 (ref)		1 (ref)	
> 7.1	2.88 (2.69–3.08)	< 0.001	1.41 (1.30–1.54)	< 0.001
Prothrombin time, s				
≤ 16	1 (ref)		1 (ref)	
> 16	3.45 (3.11–3.85)	< 0.001	1.49 (1.33–1.67)	< 0.001
D-dimer, mg/L				
< 0.5	1 (ref)		1 (ref)	
0.5–1	1.23 (1.14–1.34)	< 0.001	1.03 (0.96–1.12)	0.41
> 1	1.54 (1.42–1.66)	< 0.001	1.03 (0.95–1.11)	0.50
Hyponatremia	1.57 (1.43–1.73)	< 0.001	1.09 (0.99–1.21)	0.07
Hypernatremia	2.38 (2.13–2.67)	< 0.001	1.34 (1.19–1.51)	< 0.001
Hypokalemia	1.24 (1.13–1.36)	< 0.001	1.11 (1.01–1.22)	0.03
Hyperkalemia	2.61 (2.20–3.11)	< 0.001	1.23 (1.03–1.48)	0.02
Hypercholesterolemia	1.37 (1.26–1.49)	< 0.001	1.08 (1.01–1.16)	0.02
Hypertriglyceridemia	1.10 (1.01–1.20)	0.03	0.94 (0.86–1.03)	0.22
Underlying disease				
Hypertension	1.22 (1.12–1.32)	< 0.001		
Diabetes	1.20 (1.08–1.34)	< 0.001		
COPD	1.24 (1.01–1.53)	< 0.001		
ARDS	6.34 (5.60–7.17)	< 0.001	2.47 (2.16–2.83)	< 0.001
Coronary heart disease	1.26 (1.12–1.44)	< 0.001		
Arrhythmia	1.17 (0.97–1.42)	0.10		
Heart failure	2.72 (2.40–3.10)	< 0.001	1.06 (0.93–1.22)	0.37
Chronic gastritis	0.42 (0.31–0.58)	< 0.001		
Gastric ulcer	0.60 (0.25–1.44)	0.25		
Cirrhosis	1.74 (1.17–2.60)	0.007		
Hyperthyroidism	0.63 (0.25–1.52)	0.31		
Chronic renal failure	4.26 (3.77–4.82)	< 0.001		
Stroke	1.23 (1.08–1.40)	0.001		
Rheumatoid arthritis	0.94 (0.45–1.98)	0.87		
SLE	0.73 (0.18–2.90)	0.65		
Solid tumor	1.89 (1.57–2.28)	< 0.001	1.10 (0.91–1.33)	0.34
Hematological tumor	3.30 (2.05–5.31)	< 0.001	3.02 (1.87–4.89)	< 0.001
Schizophrenia	0.17 (0.10–0.30)	< 0.001		
Depression	0.41 (0.22–0.77)	0.005		
Sepsis	5.96 (5.10–6.97)	< 0.001		
Shock	10.02 (8.93–11.24)	< 0.001	3.02 (2.66–3.44)	< 0.001
Medicine				
ACEI	2.69 (2.28–3.18)	< 0.001	1.05 (0.88–1.25)	0.57
ARB	0.73 (0.61–0.89)	0.001	0.57 (0.47–0.69)	< 0.001
β receptor blockers	1.29 (1.15–1.44)	< 0.001		
CCB	1.11 (1.03–1.21)	0.04		
Diuretic	4.46 (4.16–4.77)	< 0.001		
ARNI	1.53 (0.58–4.10)	0.39		
Digitalis	6.93 (6.10–7.87)	< 0.001		
Hydroxychloroquine	0.47 (0.35–0.63)	0.001		
Insulin	3.83 (3.54–4.13)	< 0.001	3.35 (3.08–3.64)	< 0.001
Metformin	0.45 (0.34–0.58)	< 0.001	0.26 (0.20–0.34)	< 0.001
Sulfonylureas	0.54 (0.37–0.77)	< 0.001		
AGI	0.58 (0.41–0.72)	< 0.001		
Antiplatelet drugs	1.23 (1.09–1.40)	0.001		
Anticoagulant	4.17 (3.88–4.48)	< 0.001		
Statins	1.04 (0.92–1.19)	0.60	0.69 (0.60–0.80)	< 0.001
Nitrates	3.66 (3.31–4.05)	< 0.001		
Corticosteroid	2.75 (2.55–2.96)	< 0.001		
IVIG	2.81 (2.62–3.01)	< 0.001		

Keys: SLE, systemic lupus erythematosus; ACEI, angiotensin-converting enzyme inhibitors; ARB, angiotensin II receptor blockers; CCB, Calcium channel blockers; ARNI, angiotensin receptor-neprilysin inhibitors; AGI, alpha-glucosidase inhibitors; IVIG, intravenous immunoglobulin.

### 3.4 Survival analysis of different sex, age and severity

**Fig. 4:**
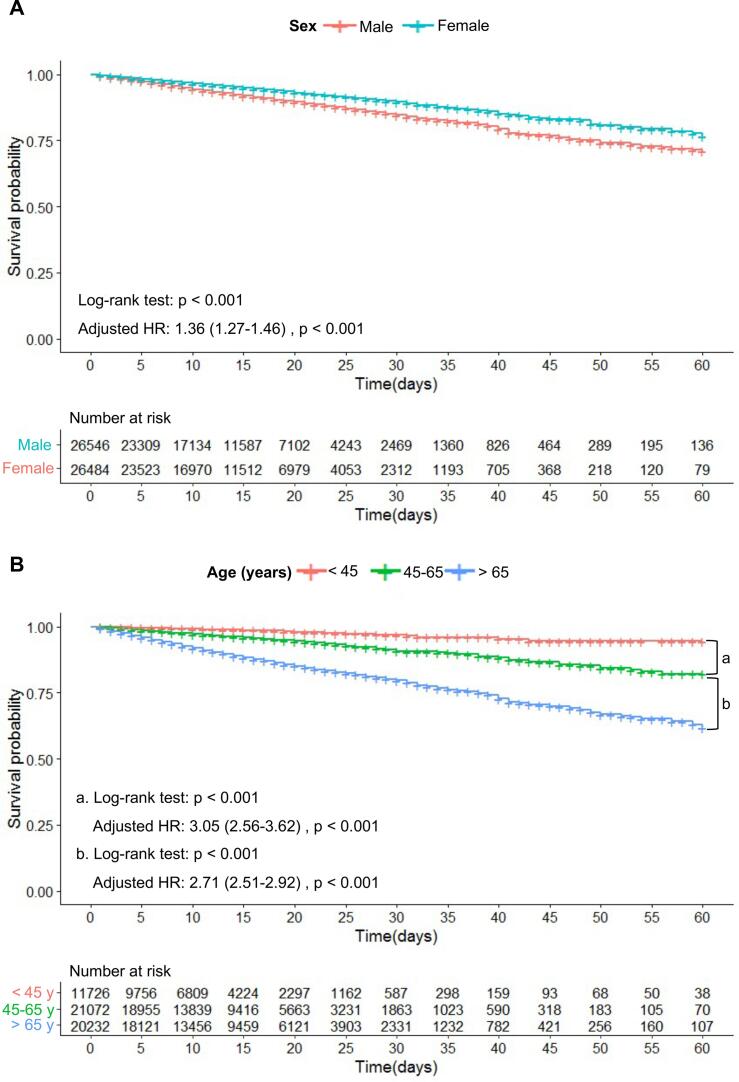
The Kaplan–Meier survival curve of in-hospital mortality for COVID-19 patients of different sex (A) and age (B).

We conducted a survival analysis of patients with COVID-19 of various sexes, ages, and severity. The Kaplan–Meier survival curve and multivariate Cox regression analyses (none of which violated the proportional hazard hypothesis) between different age groups showed that male patients had a significantly higher in-hospital mortality (log-rank P < 0.001; adjusted HR, 1.36; 95 % CI, 1.27–1.46; P < 0.001) than female patients (Fig. 4A). Survival analysis among different age groups (<45, 45–65, and > 65 years) indicated that mortality increased significantly with age (Fig. 4B). The in-hospital mortality of patients aged 45–65 years was significantly higher than that of patients aged < 45 years (log-rank P < 0.001; adjusted HR, 3.05; 95 % CI, 2.56–3.62; P < 0.001). However, the in-hospital mortality of patients aged > 65 years was higher than that of patients aged 45–65 years (log-rank P < 0.001; adjusted HR, 2.71; 95 % CI, 2.51–2.92; P < 0.001).

**Fig. 5:**
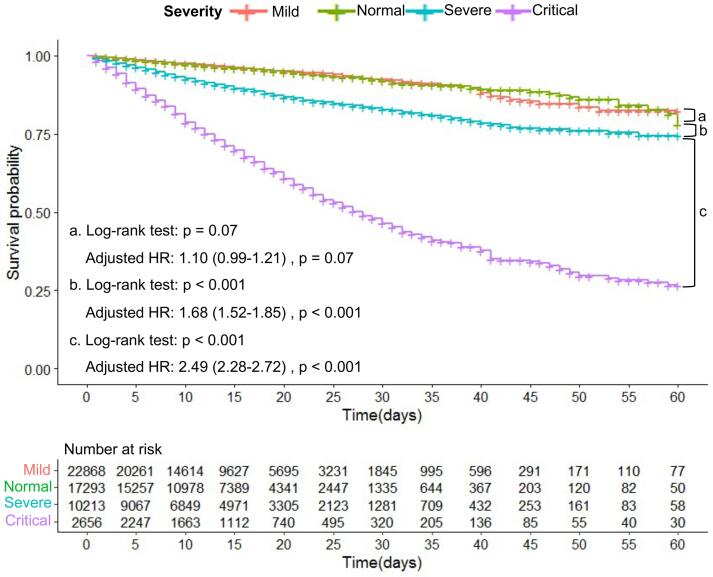
The Kaplan–Meier survival curve of in-hospital mortality for COVID-19 patients of different severity.

In addition, we compared the in-hospital mortality rates of patients with COVID-19 with differing disease severity levels (Fig. 5). The Kaplan–Meier survival curve and multivariate Cox regression analyses showed that in-hospital mortality increased with increasing severity (Severe vs. Moderate: log-rank P < 0.001; adjusted HR, 1.68; 95 % CI, 1.52–1.85; P < 0.001; Critical vs. Severe: log-rank P < 0.001; adjusted HR, 2.49; 95 % CI, 2.28–2.72; P < 0.001), although there was no statistically significant difference between healthy and mild patients (Moderate vs. Mild: log-rank P = 0.07; adjusted HR, 1.10; 95 % CI, 0.99–1.21; P = 0.07).

## 4 Discussion

In this multicenter retrospective study, we examined the clinical characteristics and mortality risk factors of hospitalized patients from the earliest COVID-19 outbreak based on 138 hospital records from Hubei Province. The mortality rate of all hospitalized COVID-19 patients was approximately 7.0 %. We found that male sex, age > 65 years, DBP > 90 mmHg, WBC > 10 × 10^9^ /L, blood glucose > 6.1 mmol/L, ALT > 40 U/L, LDH > 245 U/L, Cr > 133 μmol/L, BUN > 7.1 mmol/L, and PT > 16 s as well as hyponatremia, hypokalemia, ARDS, shock, solid tumors, hematological tumors, and insulin use were potential risk factors for in-hospital mortality in patients with COVID-19. In addition, there were significant differences among the survival analysis of patients with COVID-19 based on sex, age, and severity. Males aged > 65 years and critically ill patients with COVID-19 had a lower survival rate.

In our study, cardiovascular disease was the most common comorbidity in patients with COVID-19, followed by endocrine and digestive diseases. These results showed that most patients with COVID-19 had initial comorbidities and should receive special assistance. According to a study, hypertension, obesity, and diabetes were the most common comorbid conditions among 5700 patients with COVID-19 in the New York City area[13], which was similar to our results. In patients with comorbidities, SARS-CoV-2 infection can become more detrimental and deadly, requiring meticulous management of patients with COVID-19 with comorbidities to avoid the risk of deat[14].

With increasing age, patients with COVID-19 seem to have a deteriorating prognosis and higher mortality[15], [16]. Aging is considered a significant independent risk factor for death among patients with COVID-19[17]. Additionally, older age (especially > 65 years) was found to be associated with higher mortality and was an independent risk factor of death in patients with COVID-19, which might be due to early comorbidities, immunosenescence, inflammation, or coagulation-aging[17], [18], [19]. Furthermore, our study demonstrated that the male sex is associated with an increased risk of death after accounting for age, severity, and other underlying conditions. In similar studies, the male sex has been demonstrated to be an independent risk factor for multiple endpoint events in patients with COVID-19[7], [20], [21]. In addition, we found that some comorbidities and abnormal laboratory values contributed to COVID-19 mortality, similarly to the current mainstream perception[22], [23]. We also analyzed the risk factors of mortality in patients with non-severe and severe COVID-19. These results were consistent with the overall risk factors and confirmed some of the risk factors identified in previous studies[6], [24], [25].In the end, our study identifies key risk groups that warrant particular attention: elderly patients (over 65 years old), those with pre-existing cardiovascular conditions, and those with metabolic diseases. These groups are shown to have increased vulnerability to severe COVID-19 outcomes. Based on these findings, we suggest prioritizing rigorous vaccination efforts for these high-risk groups and implementing strict clinical monitoring following infection to manage and mitigate the long-term cardiovascular effects of COVID-19. We believe that these insights will be significant for optimizing patient care and public health strategies, especially in protecting vulnerable populations from severe outcomes and potential cardiovascular sequelae following COVID-19 infection.

The previous studies were mostly retrospective studies of relatively small populations in a single hospital or a few hospitals, with some bias in data selection[2], [16], [26], [27], [28]. This study included clinical data on patients with COVID-19 from 138 hospitals in Hubei Province, which could confirm and complement previous findings and be used to develop and validate clinical risk-scoring models[29]. We systematically analyzed the clinical characteristics and risk factors for in-hospital mortality in adult patients with COVID-19 from 2019 to 2021 in Hubei Province. These findings can help us assess the progress of COVID-19 and provide guidance for clinical intervention strategies for patients with COVID-19 or other acute infectious diseases.

## 5 Authors’ contribution

WH and GL designed the study, collected and analyzed the data, performed the statistical analysis, and wrote the draft of the manuscript. KX, YY, BY, YS, JW, KZ, and DZ completed the data collection and was responsible for quality control. DWW designed the study, edited the manuscript, and supervised the study. All authors gave final approval and agreed to be accountable for all aspects of the work, ensuring integrity and accuracy.

## 6 Sources of funding

This work was supported in part by the projects of National Natural Science Foundation of China (Nos. 82100526, 82,241,034 and 82330010) and National Key R&D Program of China (2024YFC3044500).

## 7 Trial registration number

ClinicalTrials.govNCT05615792.

## CRediT authorship contribution statement

**Wu He:** Writing – original draft, Visualization, Validation, Software, Methodology, Investigation, Data curation, Conceptualization. **Gen Li:** Writing – original draft, Software, Investigation, Formal analysis, Conceptualization. **Ke Xu:** Validation, Software, Resources, Formal analysis. **Bo Yu:** Visualization, Validation, Methodology, Investigation. **Yang Sun:** Validation, Software, Investigation, Conceptualization. **Kaineng Zhong:** Software, Methodology, Investigation, Formal analysis. **Da Zhou:** Validation, Supervision, Software, Methodology, Investigation, Data curation. **Yongcui Yan:** Project administration, Methodology. **Junfang Wu:** Software, Resources, Project administration. **Dao Wen Wang:** Writing – review & editing, Visualization, Validation, Resources, Methodology, Investigation, Funding acquisition, Data curation, Conceptualization.

## Declaration of competing interest

The authors declare that they have no known competing financial interests or personal relationships that could have appeared to influence the work reported in this paper.
