# Supplementary material for: Clinical characteristics and risk factors for in-hospital mortality of COVID-19 patients in Hubei Province: A multicenter retrospective study
Source: Int J Cardiol Heart Vasc. 2024 Nov 30;56:101574. doi: 10.1016/j.ijcha.2024.101574 (PMC11648888; doi:10.1016/j.ijcha.2024.101574)
Supplement: Supplementary Data 1 [file mmc1.docx]

**Supplementary Table 1. Clinical characteristics of non-severe patients with COVID-19.**

|  | | **All patients (n=40161)** | **Survivors (n=38573)** | **Non-survivors (n=1588)** | **P** |
| --- | --- | --- | --- | --- | --- |
| Gender: Male (%) | | 19776 (49.2) | 18826 (48.8) | 950 (59.8) | <0.001 |
| Age, years | | 57 (44-68) | 57 (43-67) | 71 (63-80) | <0.001 |
| Age ≥65 years (%) | | 13172 (32.8) | 12059 (31.3) | 1113 (70.1) | <0.001 |
| Age＜65 years (%) | | 26989 (67.2) | 26514 (68.7) | 475 (29.9) | <0.001 |
| Hospitalization time (days) | | 13 (7-19) | 13 (7-20) | 9 (4-16) | <0.001 |
| Severity (%) | |  |  |  |  |
| Mild | | 22868 (56.9) | 21981 (57.0) | 887 (55.9) | 0.387 |
| Moderate | | 17293 (43.1) | 16592 (43.0) | 701 (44.1) | 0.387 |
| **Vital signs** |  | | | | |
| Systolic blood pressure (mmHg) | | 121 (106-133) | 121 (106-133) | 120 (94-132) | 0.001 |
| Diastolic blood pressure (mmHg) | | 80 (72-94) | 80 (72-94) | 80 (71-97) | 0.198 |
| Respiratory rate (times per minute) | | 20 (19-20) | 20 (19-20) | 20 (19-21) | <0.001 |
| Pulse rate (times per minute) | | 80 (75-89) | 80 (75-88) | 82 (76-95) | <0.001 |
| Temperature (°C) | | 36.5 (36.3-36.8) | 36.5 (36.3-36.8) | 36.7 (36.4- 6.8) | <0.001 |
| **Original comorbidities (%)** |  | | | | |
| Hypertension (%) | | 6767 (16.8) | 6443 (16.7) | 324 (20.4) | <0.001 |
| Diabetes (%) | | 3334 (8.3) | 3181 (8.2) | 153 (9.6) | 0.055 |
| Hyperlipidemia (%) | | 20695 (51.5) | 19761 (51.2) | 934 (58.8) | <0.001 |
| Coronary heart disease (%) | | 2074 (5.2) | 1977 (5.1) | 97 (6.1) | 0.094 |
| COPD (%) | | 761 (1.9) | 726 (1.9) | 35 (2.2) | 0.408 |
| Heart failure (%) | | 834 (2.1) | 741 (1.9) | 93 (5.9) | <0.001 |
| Arrhythmia (%) | | 878 (2.2) | 835 (2.2) | 43 (2.7) | 0.173 |
| CKD (%) | | 647 (1.6) | 524 (1.4) | 123 (7.7) | <0.001 |
| Solid tumor (%) | | 665 (1.7) | 604 (1.6) | 61 (3.8) | <0.001 |
| Hematological tumor (%) | | 53 (0.1) | 47 (0.1) | 6 (0.4) | 0.018 |
| **Laboratory results** |  | | | | |
| **Routine blood test** |  | | | | |
| Red blood cell count, ×10^12^/L | | 4.19 (3.80-4.58) | 4.19 (3.81-4.58) | 4.11 (3.65-4.51) | <0.001 |
| White cell count, ×10^9^/L | | 5.77 (4.60-7.40) | 5.75 (4.59-7.34) | 6.57 (4.97-9.30) | <0.001 |
| Hemoglobin g/L | | 127 (115-139) | 127 (115-139) | 124 (109-137) | <0.001 |
| Neutrophil count, ×10^9^/L | | 3.57 (2.67-4.99) | 3.55 (2.66-4.94) | 4.48 (3.06-7.59) | <0.001 |
| Monocyte count, ×10^9^/L | | 0.41 (0.30-0.54) | 0.41 (0.30-0.54) | 0.41 (0.30-0.56) | 0.493 |
| Platelet count, ×10^9^/L | | 202 (162-241) | 203 (163-242) | 188 (139-236) | <0.001 |
| **Blood biochemistry** |  | | | | |
| Alanine aminotransferase, U/L | | 21 (14-35) | 21 (14-35) | 23 (15-39) | <0.001 |
| Aspartate aminotransferase, U/L | | 22 (17-31) | 22 (17-30) | 26 (19-41) | <0.001 |
| Lactate dehydrogenase, U/L | | 187 (155-240) | 186 (154-238) | 221 (166-365) | <0.001 |
| Total bilirubin, umol/L | | 10.10 (7.40-13.80) | 10.08 (7.40-13.80) | 10.53 (7.80-15.01) | <0.001 |
| Total protein, g/L | | 67.20 (62.50-71.90) | 67.20 (62.60-72.00) | 66.00 (60.50-70.70) | <0.001 |
| Globulin, g/L | | 27.90 (24.40-31.70) | 27.80 (24.40-31.60) | 28.90 (25.00-33.40) | <0.001 |
| Albumin, g/L | | 39.10 (35.10-42.90) | 39.20 (35.20-43.00) | 36.35 (31.30-40.90) | <0.001 |
| Alkaline phosphatase, U/L | | 67 (53-84) | 67 (53-84) | 70 (55-90) | <0.001 |
| Total cholesterol, mmol/L | | 4.14 (3.48-4.88) | 4.15 (3.49-4.89) | 3.88 (3.23-4.63) | <0.001 |
| Triglyceride, mmol/L | | 1.30 (0.96-1.85) | 1.30 (0.96-1.85) | 1.33 (0.96-1.96) | 0.076 |
| LDL, mmol/L | | 2.44 (1.93-3.04) | 2.44 (1.93-3.05) | 2.28 (1.74-2.86) | <0.001 |
| HDL, mmol/L | | 1.08 (0.89-1.31) | 4.15 (3.49-4.89) | 3.88 (3.23-4.63) | <0.001 |
| Creatinine, μmol/L | | 66.00 (55.00-80.00) | 66.00 (54.90-80.00) | 72.05 (57.85-92.00) | <0.001 |
| Blood urea nitrogen, mmol/L | | 4.50 (3.57-5.80) | 4.50 (3.56-5.77) | 5.24 (3.90-8.02) | <0.001 |
| eGFR, mL/min | | 100.10 (86.80-114.70) | 100.30 (87.10-114.90) | 95.10 (73.30-110.62) | <0.001 |
| Sodium, mmol/L | | 140.00 (137.50-141.90) | 140.00 (137.60-141.80) | 139.50 (136.60-142.00) | 0.018 |
| Potassium, mmol/L | | 4.01 (3.70-4.35) | 4.01 (3.70-4.35) | 4.03 (3.66-4.42) | 0.189 |
| Calcium, mmol/L | | 2.24 (2.12-2.37) | 2.24 (2.12-2.37) | 2.17 (2.04-2.33) | <0.001 |
| Lactic acid, mmol/L | | 1.70 (1.20-2.30) | 1.70 (1.20-2.30) | 1.90 (1.40-2.57) | <0.001 |
| **Coagulation function** | |  |  |  |  |
| APTT, s | | 31.70 (27.80-36.30) | 31.20 (27.50-35.20) | 32.45 (28.17-37.73) | <0.001 |
| Prothrombin time, s | | 12.50 (11.10-13.70) | 12.30 (11.00-13.50) | 12.70 (11.30-14.20) | <0.001 |
| Prothrombin activity, % | | 95.00 (84.00-106.00) | 96.20 (86.00-107.00) | 92.00 (78.90-103.00) | <0.001 |
| Thrombin time, s | | 16.50 (15.30-17.90) | 16.50 (15.20-17.80) | 16.60 (15.30-18.10) | <0.001 |
| International normalized ratio | | 1.02 (0.93-1.12) | 1.00 (0.91-1.10) | 1.05 (0.94-1.16) | <0.001 |
| Fibrinogen, mg/L | | 3.36 (2.62-4.36) | 3.30 (2.56-4.27) | 3.56 (2.65-4.60) | <0.001 |
| D-dimer, mg/L | | 0.54 (0.29-1.03) | 0.44 (0.23-0.84) | 0.59 (0.29-1.12) | <0.001 |
| **Cardiac function related indicators** |  | | | | |
| NT-proBNP, pg/mL | | 95.00 (32.23-308.20) | 92.10 (32.00-297.00) | 198.76 (55.79-919.62) | <0.001 |
| hscTnI, , pg/mL | | 0.04 (0.01-3.60) | 0.04 (0.01-3.60) | 0.07 (0.01-5.31) | <0.001 |
| CK-MB, U/L | | 7.00 (0.90-12.10) | 7.00 (0.90-12.00) | 8.00 (1.30-14.00) | <0.001 |
| **Diabetes related index** |  | | | | |
| Glucose, mmol/L | | 5.63 (4.98-7.00) | 5.61 (4.98-6.95) | 6.14 (5.14-8.43) | <0.001 |
| HbA1c, % | | 6.20 (5.70-7.20) | 6.20 (5.70-7.20) | 6.20 (5.70-7.10) | 0.855 |
| **Treatment** |  | | | | |
| Corticosteroids | | 2724 (6.8) | 2339 (6.1) | 385 (24.2) | <0.001 |
| Intravenous immunoglobin | | 3530 (8.8) | 3085 (8.0) | 445 (28.0) | <0.001 |
| Mechanical ventilation | | 844 (2.1) | 412 (1.1) | 432 (27.2) | <0.001 |
| ECMO | | 131 (0.3) | 114 (0.3) | 17 (1.1) | <0.001 |
| CRRT | | 202 (0.5) | 134 (0.3) | 68 (4.3) | <0.001 |
| **Complications** |  | | | | |
| Sepsis (%) | | 203 (0.5) | 131 (0.3) | 72 (4.5) | <0.001 |
| ARDS (%) | | 269 (0.7) | 163 (0.4) | 106 (6.7) | <0.001 |
| Thrombotic complications (%) | | 2009 (5.00) | 1891 (4.90) | 118 (7.43) | <0.001 |

Keys: COPD, chronic obstructive pulmonary disease; CKD, chronic kidney disease; LDL, low-density lipoprotein; HDL, high-density lipoprotein; eGFR, estimated glomerular filtration rate; APTT, activated partial thromboplastin time; NT-proBNP, N-terminal pro-B-type natriuretic peptide; hscTnI, high-sensitivity cardiac troponin I; CK-MB, creatine kinase-MB isoenzyme; ECMO, extracorporeal membrane oxygenation; CRRT, continuous renal replacement therapy; ARDS, acute respiratory distress syndrome.

**Supplementary Table 2. Clinical characteristics of severe patients with COVID-19.**

|  | | **All patients (n=12869)** | **Survivors (n=10747)** | **Non-survivors (n=2122)** | **P** |
| --- | --- | --- | --- | --- | --- |
| Gender: Male (%) | | 6770 (52.6) | 5456 (50.8) | 1314 (61.9) | <0.001 |
| Age, years | | 66 (56-76) | 65 (54-75) | 71 (63-80) | <0.001 |
| Age ≥65 years (%) | | 7060 (54.9) | 5563 (51.8) | 1497 (70.5) | <0.001 |
| Age＜65 years (%) | | 5809 (45.1) | 5184 (48.2) | 625 (29.5) | <0.001 |
| Hospitalization time (days) | | 14 (7-22) | 15 (8-23) | 9 (5-17) | <0.001 |
| Severity (%) | |  |  |  |  |
| Severe | | 10213 (79.4) | 9085 (84.5) | 1128 (53.2) | <0.001 |
| Critical | | 2656 (20.6) | 1662 (15.5) | 994 (46.8) | <0.001 |
| **Vital signs** |  | | | | |
| Systolic blood pressure (mmHg) | | 122 (103-133) | 122 (105-134) | 120 (91-132) | 0.001 |
| Diastolic blood pressure (mmHg) | | 80 (71-93) | 80 (71-92) | 80 (71-98) | 0.057 |
| Respiratory rate (times per minute) | | 20 (19-20) | 20 (19-20) | 20 (19-21) | <0.001 |
| Pulse rate (times per minute) | | 80 (75-89) | 80 (75-88) | 84 (76-95) | <0.001 |
| Temperature (°C) | | 36.50 (36.30-36.80) | 36.50 (36.0-36.80) | 36.70 (36.40-36.80) | <0.001 |
| **Original comorbidities (%)** |  | | | | |
| Hypertension (%) | | 2969 (23.1) | 2534 (23.6) | 435 (20.5) | <0.001 |
| Diabetes (%) | | 1588 (12.3) | 1354 (12.6) | 234 (11.0) | 0.048 |
| Hyperlipidemia (%) | | 7018 (54.5) | 5714 (53.2) | 1304 (61.5) | <0.001 |
| Coronary heart disease (%) | | 1139 (8.9) | 976 (9.1) | 163 (7.7) | 0.042 |
| COPD (%) | | 403 (3.1) | 345 (3.2) | 58 (2.7) | 0.278 |
| Heart failure (%) | | 584 (4.5) | 427 (4.0) | 157 (7.4) | <0.001 |
| Arrhythmia (%) | | 475 (3.7) | 409 (3.8) | 66 (3.1) | 0.136 |
| CKD (%) | | 398 (3.1) | 248 (2.3) | 150 (7.1) | <0.001 |
| Solid tumor (%) | | 305 (2.4) | 253 (2.4) | 52 (2.5) | 0.85 |
| Hematological tumor (%) | | 28 (0.2) | 17 (0.2) | 11 (0.5) | 0.003 |
| **Laboratory results** |  | | | | |
| **Routine blood test** |  | | | | |
| Red blood cell count, ×10^12^/L | | 4.12 (3.71-4.53) | 4.13 (3.73-4.53) | 4.10 (3.64-4.52) | 0.002 |
| White cell count, ×10^9^/L | | 6.04 (4.70-7.95) | 5.94 (4.65-7.72) | 6.78 (5.04-9.91) | <0.001 |
| Hemoglobin g/L | | 125 (112-137) | 125 (112-137) | 123 (109-137) | <0.001 |
| Neutrophil count, ×10^9^/L | | 3.91 (2.84-5.76) | 3.77 (2.77-5.43) | 4.74 (3.28-8.36) | <0.001 |
| Monocyte count, ×10^9^/L | | 0.41 (0.30-0.55) | 0.41 (0.30-0.55) | 0.40 (0.28-0.54) | 0.493 |
| Platelet count, ×10^9^/L | | 195 (153-236) | 197 (157-238) | 181 (129-224) | <0.001 |
| **Blood biochemistry** |  | | | | |
| Alanine aminotransferase, U/L | | 22 (14-37) | 22 (14-36) | 24 (15-41) | <0.001 |
| Aspartate aminotransferase, U/L | | 23 (17-34) | 23 (17-32) | 24 (15-41) | <0.001 |
| Lactate dehydrogenase, U/L | | 199 (161-277) | 195 (159-261) | 244 (176-391) | <0.001 |
| Total bilirubin, umol/L | | 10.20 (7.40-14.20) | 10.10 (7.36-14.00) | 10.80 (7.80-15.80) | <0.001 |
| Total protein, g/L | | 66.40 (61.40-71.30) | 66.50 (61.60-71.40) | 65.60 (60.20-71.00) | <0.001 |
| Globulin, g/L | | 28.30 (24.60-32.40) | 28.10 (24.50-32.10) | 29.35 (25.20-33.60) | <0.001 |
| Albumin, g/L | | 37.60 (33.50-41.74) | 38.00 (34.00-42.00) | 35.60 (30.80-40.30) | <0.001 |
| Alkaline phosphatase, U/L | | 69 (55-88) | 69 (55-87) | 72 (56-94) | <0.001 |
| Total cholesterol, mmol/L | | 4.04 (3.39-4.80) | 4.10 (3.44-4.84) | 3.81 (3.13-4.55) | <0.001 |
| Triglyceride, mmol/L | | 1.30 (0.96-1.86) | 1.29 (0.95-1.85) | 1.36 (0.99-1.91) | 0.076 |
| LDL, mmol/L | | 2.44 (1.93-3.04) | 2.41 (1.90-3.01) | 2.21 (1.69-2.83) | <0.001 |
| HDL, mmol/L | | 1.05 (0.86-1.29) | 1.06 (0.87-1.30) | 0.98 (0.79-1.21) | <0.001 |
| Creatinine, μmol/L | | 67.00 (55.30-83.00) | 66.70 (55.00-81.50) | 70.05 (57.00-91.00) | <0.001 |
| Blood urea nitrogen, mmol/L | | 4.80 (3.70-6.40) | 4.68 (3.69-6.10) | 5.60 (4.10-8.60) | <0.001 |
| eGFR, mL/min | | 98.00 (83.00-112.60) | 98.60 (84.40-113.00) | 95.19 (74.00-110.78) | <0.001 |
| Sodium, mmol/L | | 139.50 (137.00-141.70) | 139.50 (137.00-141.60) | 139.40 (136.80-142.00) | 0.868 |
| Potassium, mmol/L | | 4.03 (3.70-4.38) | 4.02 (3.70-4.37) | 4.05 (3.70-4.44) | 0.037 |
| Calcium, mmol/L | | 2.22 (2.09-2.35) | 2.23 (2.11-2.35) | 2.15 (2.00-2.31) | <0.001 |
| Lactic acid, mmol/L | | 1.70 (1.30-2.40) | 1.70 (1.22-2.30) | 1.90 (1.40-2.60) | <0.001 |
| **Coagulation function** | |  |  |  |  |
| APTT | | 31.70 (27.80-36.30) | 31.50 (27.70-36.20) | 32.60 (28.50-37.30) | <0.001 |
| Prothrombin time, s | | 12.50 (11.10-13.70) | 12.40 (11.10-13.60) | 13.00 (11.50-14.30) | <0.001 |
| Prothrombin activity, % | | 95.00 (84.00-106.00) | 96.00 (85.00-106.20) | 91.00 (77.93-102.00) | <0.001 |
| Thrombin time, s | | 16.50 (15.30-17.90) | 16.50 (15.30-17.90) | 16.60 (15.30-18.00) | 0.677 |
| International normalized ratio | | 1.02 (0.93-1.12) | 1.01 (0.92-1.11) | 1.06 (0.96-1.18) | <0.001 |
| Fibrinogen, mg/L | | 3.36 (2.62-4.36) | 3.34 (2.62-4.32) | 3.45 (2.62-4.56) | <0.001 |
| D-dimer, mg/L | | 0.54 (0.29-1.03) | 0.53 (0.28-1.00) | 0.59 (0.29-1.18) | <0.001 |
| **Cardiac function related indicators** |  | | | | |
| NT-proBNP, pg/mL | | 124.00 (39.30, 463.54) | 114.30 (37.55, 402.40) | 206.55 (55.37, 1083.29) | <0.001 |
| hscTnI, pg/mL | | 0.05 (0.01, 4.20) | 0.05 (0.01, 4.00) | 0.12 (0.01, 5.90) | <0.001 |
| CK-MB, U/L | | 7.40 (1.00, 13.00) | 7.00 (0.99, 13.00) | 8.90 (1.60, 15.00) | <0.001 |
| **Diabetes related index** |  | | | | |
| Glucose, mmol/L | | 5.85 (5.08-7.53) | 5.77 (5.04-7.28) | 6.44 (5.27-9.02) | <0.001 |
| HbA1c, % | | 6.20 (5.70-7.30) | 6.20 (5.70-7.30) | 6.30 (5.70-7.30) | 0.065 |
| **Treatment** |  | | | | |
| Corticosteroids | | 1576 (12.2) | 1057 (9.8) | 519 (24.5) | <0.001 |
| Intravenous immunoglobin | | 2100 (16.3) | 1405 (13.1) | 695 (32.8) | <0.001 |
| Mechanical ventilation | | 974 (7.6) | 316 (2.9) | 658 (31.0) | <0.001 |
| ECMO | | 65 (0.5) | 30 (0.3) | 35 (1.6) | <0.001 |
| CRRT | | 180 (1.4) | 60 (0.6) | 120 (5.7) | <0.001 |
| **Complications** |  | | | | |
| Sepsis (%) | | 197 (1.5) | 104 (1.0) | 93 (4.4) | <0.001 |
| ARDS (%) | | 282 (2.2) | 114 (1.1) | 168 (7.9) | <0.001 |
| Thrombotic complications (%) | | 947 (7.36) | 723 (6.73) | 224 (10.56) | <0.001 |

**Supplementary Table 3. Risk factors associated with in-hospital mortality in non-severe COVID-19 patients.**

|  | **Unadjusted** |  | **Adjusted** |  |
| --- | --- | --- | --- | --- |
| **Characteristics** | **HR (95%CI)** | **P** | **HR (95%CI)** | **P** |
| Age, years |  |  |  |  |
| <65 | 1 (ref) |  | 1 (ref) |  |
| ≥ 65 | 1.96 (1.79-2.15) | < 0.001 | 1.82 (1.65-2.00) | < 0.001 |
| Sex |  |  |  |  |
| Female | 1 (ref) |  | 1 (ref) |  |
| Male | 1.47 (1.35-1.61) | < 0.001 | 1.33 (1.22-1.45) | < 0.001 |
| Systolic blood pressure (mmHg) |  |  |  |  |
| ≤ 140 | 1 (ref) |  | 1 (ref) |  |
| > 140 | 1.07 (0.95-1.19) | 0.27 | 1.07 (0.96-1.20) | 0.25 |
| Diastolic blood pressure (mmHg) |  |  |  |  |
| ≤ 90 | 1 (ref) |  | 1 (ref) |  |
| > 90 | 1.18 (1.08-1.29) | < 0.001 | 1.05 (0.96-1.16) | 0.30 |
| White blood cell count, ×10^9^/L |  |  |  |  |
| 4-10 | 1 (ref) |  | 1 (ref) |  |
| < 4 | 0.78 (0.67-0.91) | 0.001 | 0.85 (0.74-0.99) | 0.04 |
| >10 | 2.28 (2.06-2.52) | < 0.001 | 1.34 (1.2-1.50) | < 0.001 |
| HbA1c, % |  |  |  |  |
| ≤ 6 | 1 (ref) |  | 1 (ref) |  |
| > 6 | 1.09 (1.00-1.19) | 0.06 | 0.99 (0.91-1.08) | 0.84 |
| Glucose, mmol/L |  |  |  |  |
| ≤ 6.1 | 1 (ref) |  | 1 (ref) |  |
| > 6.1 | 1.65 (1.51-1.80) | < 0.001 | 1.21 (1.11-1.33) | < 0.001 |
| Alanine aminotransferase, U/L |  |  |  |  |
| ≤ 40 | 1 (ref) |  | 1 (ref) |  |
| > 40 | 1.82 (1.65-2.00) | < 0.001 | 1.30 (1.17-1.43) | < 0.001 |
| Lactate dehydrogenase, U/L |  |  |  |  |
| ≤ 245 | 1 (ref) |  | 1 (ref) |  |
| > 245 | 2.02 (1.86-2.21) | < 0.001 | 1.37 (1.25-1.51) | < 0.001 |
| eGFR, mL/min |  |  |  |  |
| ≥ 90 | 1 (ref) |  |  |  |
| < 90 | 1.38 (1.26-1.50) | < 0.001 |  |  |
| Creatinine, μmol/L |  |  |  |  |
| ≤ 133 | 1 (ref) |  | 1 (ref) |  |
| > 133 | 2.43 (2.13-2.78) | < 0.001 | 1.15 (0.98-1.35) | 0.08 |
| Blood urea nitrogen, mmol/L |  |  |  |  |
| ≤ 7.1 | 1 (ref) |  | 1 (ref) |  |
| > 7.1 | 2.42 (2.21-3.64) | < 0.001 | 1.39 (1.24-1.55) | < 0.001 |
| Prothrombin time, s |  |  |  |  |
| ≤ 16 | 1 (ref) |  | 1 (ref) |  |
| > 16 | 2.89 (2.53-3.30) | < 0.001 | 1.43 (1.24-1.65) | < 0.001 |
| D-dimer, mg/L |  |  |  |  |
| < 0.5 | 1 (ref) |  |  |  |
| 0.5-1 | 1.01 (0.91-1.12) | 0.85 |  |  |
| > 1 | 1.18 (1.07-1.31) | 0.001 |  |  |
| Hyponatremia | 1.28 (1.13-1.46) | < 0.001 | 1.00 (0.88-1.14) | 0.99 |
| Hypernatremia | 2.28 (1.97-2.64) | < 0.001 | 1.29 (1.10-1.50) | 0.002 |
| Hypokalemia | 1.08 (0.95-1.22) | < 0.001 | 0.98 (0.87-1.11) | 0.78 |
| Hyperkalemia | 1.94 (1.54-2.44) | < 0.001 | 1.11 (0.87-1.41) | 0.40 |
| Hypercholesterolemia | 1.37 (1.26-1.49) | < 0.001 | 1.08 (0.98-1.18) | 0.11 |
| Hypertriglyceridemia | 1.11 (0.99-1.25) | 0.07 | 0.94 (0.83-1.06) | 0.29 |
| Underlying disease |  |  |  |  |
| Hypertension | 0.91 (0.82-1.01) | 0.08 |  |  |
| Diabetes | 0.93 (0.81-1.06) | 0.28 |  |  |
| COPD | 0.93 (0.71-1.20) | 0.55 |  |  |
| ARDS | 3.44 (2.93-4.02) | < 0.001 | 1.89 (1.59-2.25) | < 0.001 |
| Coronary heart disease | 0.91 (0.77-1.06) | 0.23 |  |  |
| Arrhythmia | 0.80 (0.63-1.03) | 0.08 |  |  |
| Heart failure | 1.80 (1.53-2.11) | < 0.001 | 1.08 (0.91-1.28) | 0.40 |
| Chronic gastritis | 0.30 (0.19-0.47) | < 0.001 |  |  |
| Gastric ulcer | 0.47 (0.12-1.88) | 0.26 |  |  |
| Cirrhosis | 0.95 (0.53-1.72) | 0.87 |  |  |
| Hyperthyroidism | 0.62 (0.20-1.93) | 0.41 |  |  |
| Chronic renal failure | 2.55 (2.16-3.01) | < 0.001 |  |  |
| Stroke | 0.82 (0.70-0.98) | 0.03 |  |  |
| Rheumatoid arthritis | 0.46 (0.12-1.85) | 0.27 |  |  |
| SLE | 0.56 (0.08-3.95) | 0.56 |  |  |
| Solid tumor | 1.06 (0.81-1.40) | 0.67 | 0.81 (0.62-1.08) | 0.15 |
| Hematological tumor | 2.49 (1.38-4.50) | 0.003 | 2.92 (1.61-5.31) | < 0.001 |
| Schizophrenia | 0.11 (0.05-0.27) | < 0.001 |  |  |
| Depression | 0.42 (0.18-1.02) | 0.05 |  |  |
| Sepsis | 3.06 (2.49-3.77) | < 0.001 |  |  |
| Shock | 5.11 (4.38-5.97) | < 0.001 | 2.45 (2.07-2.91) | < 0.001 |
| Medicine |  |  |  |  |
| ACEI | 1.83 (1.44-2.33) | < 0.001 | 1.03 (0.80-1.33) | 0.81 |
| ARB | 0.63 (0.49-0.82) | < 0.001 | 0.62 (0.47-0.80) | < 0.001 |
| β receptor blockers | 0.87 (0.74-1.02) | 0.09 |  |  |
| CCB | 0.90 (0.79-1.02) | 0.11 |  |  |
| Diuretic | 2.66 (2.43-2.92) | < 0.001 |  |  |
| ARNI | 1.66 (0.53-5.16) | 0.38 |  |  |
| Digitalis | 3.60 (3.01-4.30) | < 0.001 |  |  |
| Hydroxychloroquine | 0.51 (0.35-0.74) | < 0.001 |  |  |
| Insulin | 2.52 (2.27-2.79) | < 0.001 | 2.63 (2.36-2.93) | < 0.001 |
| Metformin | 0.42 (0.29-0.60) | < 0.001 | 0.28 (0.19-0.40) | < 0.001 |
| Sulfonylureas | 0.62 (0.39-0.97) | 0.04 |  |  |
| AGI | 0.43 (0.32-0.57) | < 0.001 |  |  |
| Antiplatelet drugs | 0.91 (0.76-1.08) | 0.29 |  |  |
| Anticoagulant | 2.44 (2.22-2.68) | < 0.001 |  |  |
| Statins | 0.74 (0.62-0.88) | < 0.001 | 0.63 (0.52-0.76) | < 0.001 |
| Nitrates | 2.38 (2.08-2.72) | < 0.001 |  |  |
| Corticosteroid | 1.90 (1.72-2.10) | < 0.001 |  |  |
| IVIG | 2.02 (1.85-2.22) | < 0.001 |  |  |

**Supplementary Table 4. Risk factors associated with in-hospital mortality in severe COVID-19 patients.**

|  | **Unadjusted** |  | **Adjusted** |  |
| --- | --- | --- | --- | --- |
| **Characteristics** | **HR (95%CI)** | **P** | **HR (95%CI)** | **P** |
| Age, years |  |  |  |  |
| <65 | 1 (ref) |  | 1 (ref) |  |
| >=65 | 4.23 (3.80-4.71) | < 0.001 | 3.42 (3.06-3.83) | < 0.001 |
| Sex |  |  |  |  |
| Female | 1 (ref) |  | 1 (ref) |  |
| Male | 1.52 (1.37-1.67) | < 0.001 | 1.32 (1.18-1.45) | < 0.001 |
| Systolic blood pressure (mmHg) |  |  |  |  |
| ≤ 140 | 1 (ref) |  | 1 (ref) |  |
| > 140 | 1.07 (0.94-1.22) | 0.32 | 0.98 (0.86-1.12) | 0.80 |
| Diastolic blood pressure (mmHg) |  |  |  |  |
| ≤ 90 | 1 (ref) |  | 1 (ref) |  |
| > 90 | 1.14 (1.03-1.27) | 0.01 | 1.17 (1.05-1.30) | 0.005 |
| White blood cell count, ×10^9^/L |  |  |  |  |
| 4-10 | 1 (ref) |  | 1 (ref) |  |
| < 4 | 0.89 (0.76-1.04) | 0.14 | 0.94 (0.81-1.10) | 0.45 |
| >10 | 2.91 (2.58-3.30) | < 0.001 | 1.46 (1.28-1.68) | < 0.001 |
| HbA1c, % |  |  |  |  |
| ≤ 6 | 1 (ref) |  | 1 (ref) |  |
| > 6 | 1.00 (0.91-1.11) | 0.96 | 0.94 (0.84-1.21) | 0.20 |
| Glucose, mmol/L |  |  |  |  |
| ≤ 6.1 | 1 (ref) |  | 1 (ref) |  |
| > 6.1 | 1.70 (1.54-1.87) | < 0.001 | 1.09 (0.98-1.22) | 0.10 |
| Alanine aminotransferase, U/L |  |  |  |  |
| ≤ 40 | 1 (ref) |  | 1 (ref) |  |
| > 40 | 2.05 (1.83-2.29) | < 0.001 | 1.36 (1.21-1.54) | < 0.001 |
| Lactate dehydrogenase, U/L |  |  |  |  |
| ≤ 245 | 1 (ref) |  | 1 (ref) |  |
| > 245 | 2.28 (2.06-2.52) | < 0.001 | 1.43 (1.28-1.60) | < 0.001 |
| eGFR, mL/min |  |  |  |  |
| ≥ 90 | 1 (ref) |  |  |  |
| < 90 | 1.63 (1.48-1.80) | < 0.001 |  |  |
| Creatinine, μmol/L |  |  |  |  |
| ≤ 133 | 1 (ref) |  | 1 (ref) |  |
| > 133 | 3.39 (2.89-3.98) | < 0.001 | 1.36 (1.13-1.64) | 0.001 |
| Blood urea nitrogen, mmol/L |  |  |  |  |
| ≤ 7.1 | 1 (ref) |  | 1 (ref) |  |
| > 7.1 | 2.85 (2.45-3.17) | < 0.001 | 1.37 (1.25-1.49) | < 0.001 |
| Prothrombin time, s |  |  |  |  |
| ≤ 16 | 1 (ref) |  | 1 (ref) |  |
| > 16 | 3.18 (2.66-3.80) | < 0.001 | 1.47 (1.18-1.73) | < 0.001 |
| D-dimer, mg/L |  |  |  |  |
| < 0.5 | 1 (ref) |  |  |  |
| 0.5-1 | 1.37 (1.21-1.54) | < 0.001 |  |  |
| > 1 | 1.65 (1.46-1.86) | < 0.001 |  |  |
| Hyponatremia | 1.72 (1.49-1.99) | < 0.001 | 1.21 (1.04-1.40) | 0.01 |
| Hypernatremia | 2.36 (1.98-2.81) | < 0.001 | 1.45 (1.21-1.74) | < 0.001 |
| Hypokalemia | 1.42 (1.25-1.63) | < 0.001 | 1.31 (1.15-1.50) | 0.02 |
| Hyperkalemia | 3.02 (2.31-3.94) | < 0.001 | 1.28 (0.97-1.69) | 0.09 |
| Hypercholesterolemia | 1.44 (1.30-1.58) | < 0.001 | 1.06 (0.96-1.18) | 0.26 |
| Hypertriglyceridemia | 1.09 (0.95-1.25) | 0.21 | 0.96 (0.84-1.11) | 0.59 |
| Underlying disease |  |  |  |  |
| Hypertension | 1.36 (1.20-1.53) | < 0.001 |  |  |
| Diabetes | 1.26 (1.06-1.48) | < 0.001 |  |  |
| COPD | 1.31 (0.93-1.83) | 0.12 |  |  |
| ARDS | 8.72 (7.15-10.63) | < 0.001 | 3.06 (2.47-3.80) | < 0.001 |
| Coronary heart disease | 1.34 (1.09-1.65) | 0.005 |  |  |
| Arrhythmia | 1.33 (0.99-1.81) | 0.06 |  |  |
| Heart failure | 3.07 (2.49-3.79) | < 0.001 | 0.97 (0.77-1.21) | 0.75 |
| Chronic gastritis | 0.54 (0.34-0.85) | 0.007 |  |  |
| Gastric ulcer | 0.79 (0.25-2.45) | 0.68 |  |  |
| Cirrhosis | 2.62 (1.52-4.54) | < 0.001 |  |  |
| Hyperthyroidism | 0.61 (0.15-2.45) | 0.49 |  |  |
| Chronic renal failure | 1.71 (1.46-2.01) | < 0.001 |  |  |
| Stroke | 1.51 (1.25-1.83) | < 0.001 |  |  |
| Rheumatoid arthritis | 1.57 (0.65-3.79) | 0.31 |  |  |
| SLE | 0.87 (0.12-6.19) | 0.89 |  |  |
| Solid tumor | 2.80 (2.17-3.62) | < 0.001 | 1.43 (1.09-1.86) | 0.009 |
| Hematological tumor | 3.29 (1.48-7.33) | 0.004 | 2.41 (1.07-5.43) | 0.03 |
| Schizophrenia | 0.25 (0.12-0.50) | < 0.001 |  |  |
| Depression | 0.46 (0.19-1.10) | 0.08 |  |  |
| Sepsis | 8.93 (7.04-11.32) | < 0.001 |  |  |
| Shock | 14.96 (12.58-17.79) | < 0.001 | 3.75 (3.08-4.57) | < 0.001 |
| Medicine |  |  |  |  |
| ACEI | 3.62 (2.87-4.55) | < 0.001 | 1.19 (0.93-1.52) | 0.17 |
| ARB | 0.81 (0.61-1.07) | 0.13 | 0.54 (0.40-0.72) | < 0.001 |
| β receptor blockers | 1.71 (1.46-2.01) | < 0.001 |  |  |
| CCB | 1.27 (1.11-1.46) | < 0.001 |  |  |
| Diuretic | 6.03 (5.43-6.69) | < 0.001 |  |  |
| ARNI | 0.97 (0.14-6.91) | 0.98 |  |  |
| Digitalis | 10.91 (9.11-13.08) | < 0.001 |  |  |
| Hydroxychloroquine | 0.43 (0.27-0.68) | < 0.001 |  |  |
| Insulin | 4.63 (4.11-5.21) | < 0.001 | 3.77 (3.31-4.29) | < 0.001 |
| Metformin | 0.49 (0.33-0.72) | < 0.001 | 0.26 (0.18-0.39) | < 0.001 |
| Sulfonylureas | 0.43 (0.23-0.80) | 0.008 |  |  |
| AGI | 0.73 (0.54-0.98) | 0.04 |  |  |
| Antiplatelet drugs | 1.55 (1.29-1.85) | < 0.001 |  |  |
| Anticoagulant | 5.60 (5.03-6.25) | < 0.001 |  |  |
| Statins | 1.27 (1.05-1.54) | 0.02 | 0.76 (0.62-0.93) | 0.008 |
| Nitrates | 4.59 (3.94-5.36) | < 0.001 |  |  |
| Corticosteroid | 3.22 (2.86-3.61) | < 0.001 |  |  |
| IVIG | 2.97 (2.66-3.31) | < 0.001 |  |  |
